# Misfolded G Protein-Coupled Receptors and Endocrine Disease. Molecular Mechanisms and Therapeutic Prospects

**DOI:** 10.3390/ijms222212329

**Published:** 2021-11-15

**Authors:** Alfredo Ulloa-Aguirre, Teresa Zariñán, Eduardo Jardón-Valadez

**Affiliations:** 1 Red de Apoyo a la Investigación, Universidad Nacional Autónoma de México and Instituto Nacional de Ciencias Médicas y Nutrición SZ, Mexico City 14080, Mexico; tzarinan@cic.unam.mx; 2 Departamento de Recursos de la Tierra, Universidad Autónoma Metropolitana-Lerma, Lerma de Villada 52005, Estado de México, Mexico; h.jardon@correo.ler.uam.mx

**Keywords:** G protein-coupled receptors, GPCR, protein misfolding, mutations in GPCRs, loss-of-function diseases, gonadotropin-releasing hormone receptor, GnRHR, gonadotropin receptors

## Abstract

Misfolding of G protein-coupled receptors (GPCRs) caused by mutations frequently leads to disease due to intracellular trapping of the conformationally abnormal receptor. Several endocrine diseases due to inactivating mutations in GPCRs have been described, including X-linked nephrogenic diabetes insipidus, thyroid disorders, familial hypocalciuric hypercalcemia, obesity, familial glucocorticoid deficiency [melanocortin-2 receptor, MC2R (also known as adrenocorticotropin receptor, ACTHR), and reproductive disorders. In these mutant receptors, misfolding leads to endoplasmic reticulum retention, increased intracellular degradation, and deficient trafficking of the abnormal receptor to the cell surface plasma membrane, causing inability of the receptor to interact with agonists and trigger intracellular signaling. In this review, we discuss the mechanisms whereby mutations in GPCRs involved in endocrine function in humans lead to misfolding, decreased plasma membrane expression of the receptor protein, and loss-of-function diseases, and also describe several experimental approaches employed to rescue trafficking and function of the misfolded receptors. Special attention is given to misfolded GPCRs that regulate reproductive function, given the key role played by these particular membrane receptors in sexual development and fertility, and recent reports on promising therapeutic interventions targeting trafficking of these defective proteins to rescue completely or partially their normal function.

## 1. Introduction

Proteins are macromolecule components that play key roles in the structure, function, and regulation of all living cells. Synthesis of secretory proteins begins in the endoplasmic reticulum (ER) where processing of these macromolecules is cotranslationally initiated. Both processes are tightly regulated at the transcriptional, translational, and post-translational levels by a variety of diverse signaling pathways. As proteins are synthesized, they fold and the recently synthesized protein acquires its native structure from a completely or partially unfolded state, adopting distinct conformations that yield a stable, tightly compact structure compatible with ER export. Thereafter, proteins are further processed in the Golgi to eventually continue their trafficking to their final destination within or outside the cell [1,2,3,4].

Mutations resulting in protein sequence variations may lead to errors in folding (i.e., to misfolding) due to persistent non-native interactions that affect the overall conformation of the protein and its properties in a significant manner causing different levels of malfunction in the system or systems in which these proteins are involved, leading eventually to disease [5]. Well-known diseases due to protein misfolding involve defects in ion channels, enzymes, G protein-coupled receptors (GPCRs) or other proteins, such as the tumor suppressor p53, whose gene is one of the most frequently mutated genes leading to cancer [6,7,8,9,10,11]. Disease-causing proteins that are transcribed and translated at normal levels also may be misrouted, that is, unable to reach their functional destinations in the cell or to engage the secretory pathway [2,5,12]. Protein overexpression, temperature, oxidative stress, and activation of signaling pathways associated with protein folding and quality control also may cause misfolding [13]. Misfolding and misrouting frequently results in loss-of-function of the conformationally defective protein [2,8]. Although normally some non-native interactions with other molecules may occur during the folding process (to bury highly aggregation-prone regions), conformationally abnormal proteins may aggregate provoking intra- and/or extracellular cellular protein accumulation with amyloid deposition as occurs in neurodegenerative diseases [2,14,15]. In the case of GPCRs, aggregation of misfolded proteins, as observed in neurodegenerative disorders, has been well documented only for a few misfolded GPCRs, mainly rhodopsin, in which toxic aggregates lead to retinal degeneration and disease [16,17]. In the past two decades, extraordinary efforts have been made to elucidate the molecular physiopathogenesis of diseases due to defective protein folding and to design potential therapeutic strategies that could prevent or correct the structural abnormality of disease-causing misfolded proteins [9,18,19,20,21,22,23].

This review focuses on protein misfolding and misrouting of GPCRs involved in endocrine function. Special attention is given to misfolded GPCRs that regulate reproductive function, given the key role played by these particular membrane receptors in sexual development and fertility in humans, and recent reports on promising therapeutic interventions targeting trafficking of these defective proteins to rescue completely or partially their normal function.

## 2. Loss-of-Function Diseases Caused by Folding Defects in GPCRs Associated Endocrine Function

G protein-coupled receptors are membrane receptors that vary considerably in molecular size. Nevertheless, they share a common molecular topology consisting of a single polypeptide chain of variable length that traverses the lipid bilayer forming seven characteristic transmembrane hydrophobic α-helices [transmembrane domains (TMD)], connected by alternating extracellular (EL) and intracellular (IL) sequences or loops, with an extracellular NH2-terminus or ectodomain (ECD) and an intracellular carboxyl-terminal domain or Ctail [24,25]. These receptors characteristically bind one or more heterotrimeric G proteins and other membrane associated intracellular proteins that become activated upon agonist binding, regulating activation of a variety of G protein-dependent and independent signaling cascades [26]. Activated GPCRs are rapidly desensitized and internalized via formation of endosomes, where receptor-mediated signaling continues or terminates and the fate of the internalized receptor is determined [27,28]. Thus, the net amount of a given GPCR at the plasma membrane will depend on its dynamics of intracellular export from the ER to the cell surface plasma membrane (PM), the fate of the receptor following ligand-stimulated internalization (degradation vs. recycling back to the PM), and the normal membrane turnover [28].

GPCRs are the largest family of membrane proteins in the human genome (∼4–5% of the genome codes for these proteins) and they become activated by structurally diverse ligands, which include ions, photons, odorants, lipids, and hormones and neurotransmitters that vary considerably in size, from small biogenic amines to peptides to large proteins [24]. Approximately 50% of GPCRs are receptors for endogenous ligands, whereas the remainder correspond to sensory stimuli [29]. Therefore, GPCRs are essential nodes of communication between the internal and external environments of cells, transducing the information transmitted by extracellular stimuli into intracellular signals. The large variety of endogenous and exogenous agonists that interact with GPCRs makes possible the participation of these key membrane proteins in multiple functions. Consequently, GPCRs currently represent important targets for drug design, accounting for 30–50% of sales in the pharmaceutical market [30,31].

As mentioned above, mutations resulting in changes in protein sequence may lead to misfolding, defined as a defect in protein folding due to sufficient and persistent non-native interactions that affect in a significant manner the overall architecture or three dimensional shape of the protein and/or its properties [5]. Some misfolded GPCRs retain some intrinsic functional properties, such as the capability to recognize and bind agonist and activate effectors, and thus are amenable to treatment with pharmacological chaperones (pharmacochaperones or pharmacoperones) to correct folding and misrouting, aiding the receptor to reach the PM and becoming partially or completely competent [9,21,32]. A number of endocrine diseases are associated to GPCR misfolding and loss-of-function of the abnormal receptor (Table 1); these include X-linked nephrogenic diabetes insipidus (misfolded human arginine-vassopresin 2 receptor; V2R) [33,34], thyroid disorders (thyroid-stimulating hormone receptor; TSHR) [35], familial hypocalciuric hypercalcemia (calcium-sensing receptor; CaSR) [36], obesity (melanocortin-3 and -4 receptors; MC3R and MC4R, respectively) [37,38,39,40], and familial glucocorticoid deficiency (melanocortin-2 receptor, MC2R or adrenocorticotropin receptor, ACTHR) [41]. Mutation-provoked misfolding of GPCRs involved in the regulation of reproductive function may also occur [10,42], and lead to distinct abnormalities, including hypogonadotropic hypogonadism [due to mutations in the human gonadotropin-releasing hormone receptor [43], neurokinin-3 receptor (NK3R), prokineticin receptor-2 (PROKR2), or kisspeptin receptor-1 (KISS1R) [44,45,46], male pseudohermaphroditism (due to mutations in the luteinizing hormone/chorionic gonadotropin receptor; LHCGR), and ovarian failure (mutations in the follicle-stimulating hormone receptor; FSHR) [42]. In these receptors misfolding leads to ER retention, increased intracellular degradation, and deficient trafficking of the abnormal receptor to the PM, resulting in the inability of the receptor to interact with agonists and to trigger intracellular signaling [1,47].

Because of our long-standing experience on the human GnRHR (hGnRHR) and the gonadotropin receptors, we will focus the following discussion mainly on how mutations in these GPCRs may lead to misfolding and reproductive failure.

### 2.1. Misfolded GnRHRs and Hypogonadotropic Hypogonadism

**Figure 1:**
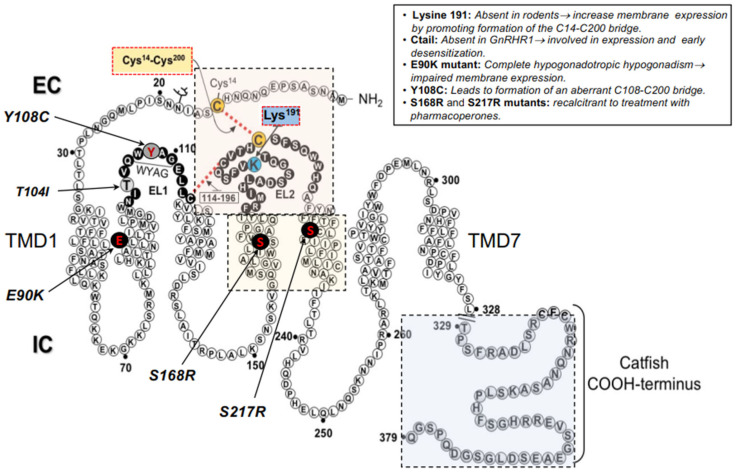
Schematic representation of the human GnRHR sequence, with circles representing amino acid residues. The red discontinuous lines within the light orange square, indicate the C14–C200 disulfide bridge, which helps to maintain the GnRHR in a conformation compatible with endoplasmic reticulum export and that is destabilized by the presence of lysine 191 (blue circle), as well as the C114–C196 bridge, which is a general structural requirement present in GPCRs belonging to the rhodopsin-like family. The light yellow square comprises the transmembrane domains (TMD) 4 and 5 which accommodates serine residues 168 and 217 that when replaced with arginine lead to severe, rescue-recalcitrant misfolding. The extracellular loop 1 (EL1) presents the conserved sequence Trp-Tyr-Ala-Gly (WYAG) [58]. In other rhodopsin-like GPCRs for peptides and biogenic amines, this sequence corresponds to the Trp-Xaa-Phe-Gly motif [(W/F)XφG, in which φ is a hydrophobic residue], which has been shown to be important for agonist-mediated receptor activation [62]. The light-blue square encompasses the sequence of the Catfish Ctail, which has been experimentally employed to test the role of the Ctail in the hGnRHR. Also indicated are the mutations E90K, Y108C, and T104I, whose effects on hGnRHR trafficking have been explored in detail [63]. The upper right inset describes some features of the mutations shown in the schematization of the receptor as well as of K191 and the Ctail, which is absent in type 1 GnRHR (see text for details).

Among the GPCRs that have served as models for understanding how alterations in the primary sequence of a GPCR may alter folding and traffic of the receptor to the PM and provoke disease as well as model for the development of pharmacological drugs to rescue function of misfolded GPCRs, is the human GnRHR [56]. This receptor is the cognate receptor for the hypothalamic decapeptide gonadotropin-releasing hormone (GnRH), which travels through the hypothalamic-pituitary portal system and binds to the GnRHR in the pituitary gonadotrops; here, GnRH stimulates the synthesis and release of the gonadotropins luteinizing hormone (LH) and follicle-stimulating hormone (FSH) [57]. The type 1 hGnRHR (hereafter referred as hGnRHR) is among the smallest members of the superfamily of GPCRs; this receptor belongs, more specifically, to the rhodopsin-like family (class A), which is the largest class of GPCRs and currently a major drug target (Table 1) [56]. The mammalian GnRHR exhibits more than 85% amino acid identity among the several species that have been cloned. Unlike other members of the rhodopsin/β-adrenergic subfamily of GPCRs, the GnRHR exhibits several unique features, including the reciprocal exchange of the conserved aspartate and asparagine residues in TMDs 2 and 7, the replacement of tyrosine with serine in the DRY motif located in the junction of the TMD 3 and the IL 2, and the lack of the intracellular Ctail [58]. This latter structural feature is not exhibited by GnRH receptors from nonmammalian vertebrate species and is unique among the thousands of members of mammalian GPCRs. Its absence in the mammalian GnRHRs is associated with differential physiological regulation including cell surface PM expression of the receptor [59,60]. In fact, it is possible to increase the PM expression levels of the hGnRHR protein by inserting the piscine Ctail DNA sequence into its corresponding *GNRHR* [59,61] (Figure 1).

Another important feature of the hGnRHR, which is closely related to receptor folding and trafficking, is the presence of a lysine residue at position 191 (K191) in the EL 2 (Figure 1). In non-primate mammals, glutamic acid or glycine (occasionally) are frequently found instead of lysine [64]; in fact, in GnRHRs from rats and mice the orthologous amino acid residue is absent, yielding a GnRHR that is one residue smaller (327 amino acid residues) than the hGnRHR [65]. Given that the absence of lysine in this position favors the proximity and interaction between the EL2 and the ectodomain of the receptor (to yield a conformation compatible with optimal ER export), the rodent GnRHRs exhibit an increased cell surface PM expression compared to that exhibited by its human counterpart [64,66,67]. In fact, K191 in the hGnRHR (328 amino acids) interferes with and limits considerably the EL2-NH_2_-terminus interaction, requiring formation of the C14–C200 bridge to stabilize the receptor in a conformation recognized by the quality control system (QCS) of the cell as correctly folded, thereby favoring trafficking of the receptor to the PM [64]. Thus, for the human GnRHR, formation of this bridge is an absolute requirement for correct routing and plasma membrane expression as bridge-breaking mutants [C200Y (a naturally occurring mutation in humans; [43,68], C14A, and C200A] exhibit either none or marginal activity [43,69,70]. Further, deletion of K191 from the hGnRHR by site-directed mutagenesis or treatment with pharmacoperones (see below) remarkably increased the cell surface plasma membrane expression of the receptor and the response to agonists [71,72]. This genetic modification (deletion of K191) and treatment with pharmacochaperones has been used in a number of misfolded GnRHR mutants as an approach to correct routing of the receptor to the PM and to restore function in in vitro and in vivo conditions [70,72,73,74].

**Figure 2:**
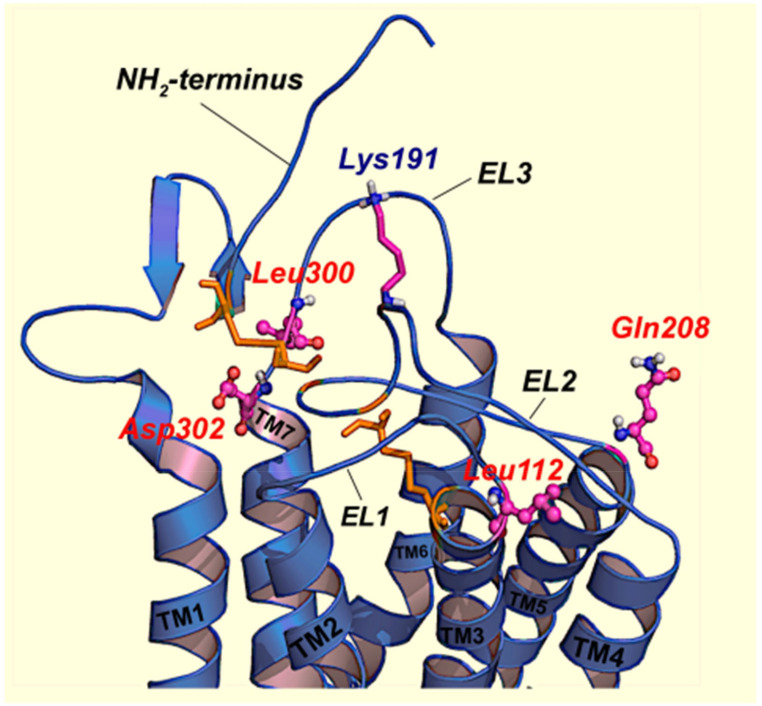
Predicted structure of the upper-third portion of the hGnRHR based on homology modeling with the structure of bovine rhodopsin [76]. The antiparallel α-helices of transmembrane domains (TM) 1 to 7 are represented by the coiled structures. These TM domains are connected by the extracellular loops (EL) of the receptor (blue curved cords). Disulfide bonds between C14 and C200 (connecting the the NH_2_-terminus and the EL2), as well as between C114 and C196 (at the COOH-terminal end of the EL1 and at the EL2) are shown as orange sticks. The location of the amino acid residues that represent a motif of four non-contiguous residues at positions L112 (at the EL1), Q208 (at the EL2), L300 (at the EL3), and D302 (at the EL3) that presumably control the destabilizing role of K191 (shown as purple, blue, and grey color sticks, at the EL2) on the association of the NH_2_-terminus and the EL2 and subsequent formation of the C14–C200 disulfide bridge, are shown in colored circles and sticks (see the text and Refs. [64,69] for data and details).

**Figure 3:**
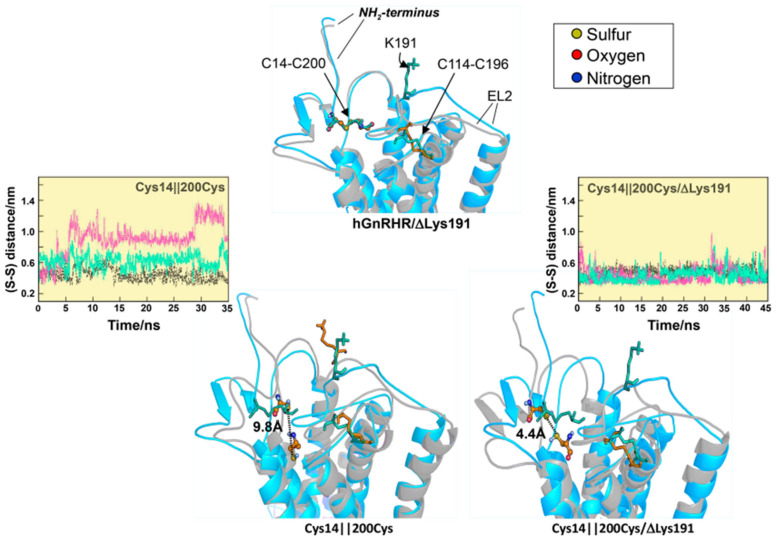
Changes in sulfur-sulfur (S-S) distances between C14 and C200 in the hGnRHR when the C14–C200 is disrupted (||) in silico, in the presence and absence of K191. Superposition of the WT hGnRHR conformation (blue structures) and the final mutant conformations (grey structures) are shown. Lysine 191 and the C14–C200 and C114–C196 disulfide bridges of the WT hGnRHR are highlighted in blue (or orange in the mutants). Residues C14 and C200 are depicted in balls and sticks. S–S distances between residues in positions 14 and 200 are also indicated (dotted lines). Insets: distances (in nm) between S-S atoms as a function of time for 3 replicas. Observe that larger average distances are found in mutants bearing K191 (for original data and details see Ref. [76]).

The evolutionary mechanisms involved in the limited PM expression of mutant and wild-type (WT) hGnRHRs imposed by the presence of K191 and the stabilizing requirement of the C14–C200 bridge have been extensively studied by combining in vitro and in silico mutational approaches [61,64,75,76]. The fact that removal of K191 obviates the requirement for the C14–C200 bridge in the hGnRHR and that inserting K191 alone into the rat or mouse GnRHR sequence (>88% homologous with the human receptor) did not impact the requirement for this bridge between the NH_2_-terminus and the EL2 [69,76] promoted the search for additional components of the requisite motif. A strategy based on identification of amino acids that both co-evolved with K191 and were thermodynamically unfavorable substitutions identified a number of motifs in multiple domains of the hGnRHR that presumably control the destabilizing influence of K191 on the formation of the C14–C200 bridge and that limit its PM expression; these include a motif of four non-contiguous residues at positions 112 (in the EL1), 208 (EL2), 300, and 302 (EL3) [64,69], that control the role of K191 on the association of the NH2-terminus and the EL2 of the hGnRHR and formation of the C14–C200 bridge essential for correct trafficking of the receptor to the PM (Figure 2). We further analyzed the role of K191 on the dynamic behavior of the hGnRHR and the formation of the C14–C200 bridge, which as previously discussed is essential for receptor trafficking to the plasma membrane [76]. Several mutants also were studied in silico: mutants lacked either the C14–C200 bridge, K191, or both. The markedly reduced expression and function of a C14S mutant lacking the C14–C200 disulfide bridge was nearly restored to WT/ΔK191 levels upon deletion of K191 [76]. Removal of K191 resulted in changes in the dynamic behavior of the mutants as revealed by molecular dynamics simulations: the distance between the sulfur- (or oxygen-) sulfur groups of cysteine (or serine) 14 and C200 was shorter and more constant, and the conformation of the NH_2_-terminus and the EL2 exhibited less fluctuations than in the presence of K191 (Figure 3). Concurrently, these biochemical and in silico data provided information on the role of K191 in defining an optimal configuration that favors hGnRHR trafficking to the PM.

Point mutations in the *GNRHR* may lead to autosomal recessive isolated hypogonadotropic hypogonadism (HH), a disease in which the pituitary response to GnRH is partially or completely affected, leading to reproductive failure [43]. Nearly 43 inactivating mutations (including deletions of large sequences and synonymous mutations) in the *GNRHR* have been described as a cause of partial or complete forms of HH [43]. Although in vitro expression of mutated human GnRHRs has shown that these mutations may impact on ligand binding and/or intracellular signaling, studies in at least half of the inactivating mutants reported to date have shown that the loss-of-function is rather due to protein misfolding causing ER retention and impaired intracellular trafficking to the PM (Class II inactivating GPCR mutations [13]; see below) [10,70]. Such ER-retained mutant GnRHRs frequently show a change in residue charge compared with the wild-type receptor (e.g., de E90K mutant), or gain or loss of either cysteine residues (known to form bridges associated with the formation of third order structure of proteins; e.g., the Y108C and C200Y GnRHRs) or proline (an amino acid residue associated with forced turns in the protein sequence, e.g., the P320L mutant GnRHR). In fact, in a number of mutant GnRHRs, function may be partially or completely restored in vitro and in vivo by deleting K191 (see above), adding a Ctail, or by exposing the cells expressing the mutant receptor to small molecule pharmacochaperones [10,23,63,72] whenever the mutation does not replace amino acid residues critical for receptor function or severely alter the three-dimensional structure of the protein. These genetic and pharmacological approaches are useful tools to analyze the molecular physiopathogenesis of HH-caused misrouted GnRHR mutants. For example, the E90K mutation, which is a typical misfolded GnRH mutant that leads to the complete form of HH [77], profoundly affects PM expression [72]. Nevertheless, either deletion of K191 (which increases membrane expression) and/or addition of the catfish Ctail (Figure 1), completely restored membrane expression and agonist-induced, receptor-mediated intracellular signaling of this mutant in vitro [10,72,78]. The Y108C mutant is also interesting (Figure 1) [79]; the fact that the amino acid residue in the second position of the WXAG motif may be highly variable (i.e., there is no consensus residue in the second position of this motif) but never involves a cysteine residue, at least in the human rhodopsin-like GPCRs studied to date [62], strongly suggests that the substitution with cysteine at this particular position (which is the case of the Y108C mutant) may profoundly alter receptor function by affecting either formation of the highly conserved EL1–EL2 disulfide bridge (C114–C196 in the hGnRHR) or the C14–C200 one. Considering that the C114–C196 bridge favors the formation of H-bonds between these loops (Figure 1) and is a structural feature associated with the fundamental stability of the many GPCRs (including the GnRHR) [80], and that its breaking leads to pharmacoperone-resistant loss of function [81], formation of a C108–114 or C108–C196 disulfide bonds seemed very unlikely. Sincethe mechanism of pharmacoperone action and the deletion of K191 is to remove the requirement (from the hGnRHR) for the C14–C200 bridge [64], we used these two experimental approaches (exposure to pharmacoperone and/or deletion of K191) to analyze in detail the molecular physiopathogenesis of HH caused by the Y108C hGnRHR mutation [63]. We found that functional recovery of a Y108C/C200A double mutant was possible indicating that blocking formation of a C108–C200 bridge resulted in a mutant that could be rescued. The observation that the Y108C/C14A double mutant could not be rescued or was only marginally rescued when both approaches were simultaneously applied was interpreted as meaning that the alanine replacement at position 14 did not protect against formation of the bridge that prevented rescue. Accordingly, we concluded that the hGnRHR mutant bearing a C108–C200 disulfide bridge was the predominant moiety formed in the Y108C mutant [63]. In contrast to these mutations, the S168R and S217R mutations (Figure 1) involve a thermodynamically unfavorable exchange that rotates the TMDs 4 and 5, moving the EL2 and making formation of the C14–C200 improbable; these latter mutants cannot pass the QCS of the cell and removal of K191 or exposure to pharmacochaperones does not rescue their function [10].

Misfolding can result in proteins that retain function but, for reasons of misrouting, cannot function normally, leading to disease. The observations that the PM expression of several misfolded, functionally competent GPCR mutants causing endocrine (Table 1) and non-endocrine diseases (e.g., retinitis pigmentosa) may be rescued by pharmacologic strategies and that several WT GPCRs whose expression is decreased in normal conditions also respond to this particular type of approach, offers a unique therapeutic opportunity for manipulating the ER QCS, both to correct misfolding defects and to improve PM expression of the WT receptor thereby increasing interaction with agonist, as it is the case of the hGnRHR and other GPCRs as well [8,82,83,84,85,86].

### 2.2. Misfolded Gonadotropin Receptors as a Cause of Hypergonadotropic Hypergonadism

The pituitary gonadotropic hormones, follicle-stimulating hormone or follitropin and luteinizing hormone or lutropin, as well as placental chorionic gonadotropin (hCG), are glycoprotein hormones that play a pivotal role in reproduction [57]. Their cognate receptors (FSHR and LHCGR—the LH receptor binds both, LH and hCG-) belong, together with the thyroid-stimulating hormone receptor (TSH), to the glycoprotein hormone receptors subfamily of the Rhodopsin-like receptors [29]. The human FSHR and LHCGR are mainly expressed by specific cells in the testes and ovaries [87,88,89]. The FSHR is expressed in the granulosa cells of the ovary and the testicular Sertoli cells of the seminiferous tubules, where it is essential for FSH-stimulated maturation of ovarian follicles and Sertoli cell growth and metabolism, promoting spermatogenesis [88,90]. In males, LHCGR is expressed in the interstitial (Leydig) cells where LH promotes production of androgens, mainly testosterone, which are converted by the Sertoli cells to estrogens [91]. In females, the LHCGR is expressed in the ovarian theca cells of the developing follicle, where LH stimulates the production of aromatizable androgens, which are subsequently biotransformed to estrogens in the granulosa cell layer [88]. A large ECD, where binding of their structurally complex ligands occurs, is a characteristic future of glycoprotein hormone receptors. This ECD is comprised of a central structural motif of imperfect leucine-rich repeats (LRR) which is shared with other membrane receptors involved in ligand selectivity and specific protein-protein interactions [92]. The carboxyl-terminal end of the large ECD exhibits a signal specificity subdomain or “hinge” region which is an integral part of the ectodomain and that links the ECD with the serpentine TMD portion of the receptors; here, activation of the receptor occurs following conformational changes provoked by the interaction of the agonist with the ECD [93,94,95]. The hinge region, whose crystal structure has been described for the human FSHR [96], has been linked to the signaling functionality of the receptor [97].

The FSHR and the LHCGR exhibit a high degree of primary sequence homology. The ECD amino acid sequences of the gonadotropin receptors are approximately 46% identical, whereas the TMDs share nearly ~70% homology [98,99]. This notable similarity between the TMDs of the gonadotropin suggests similar mechanisms of receptor activation [97]. Among the three domains, the intracellular regions have the lowest amino acid sequence homology between the FSH and LH receptors (approximately 27% identity), with the exception of the amino-terminal end of the Ctail, which exhibits cysteine residues for palmitoylation and the primary sequence motif (F(x)_6_LL) that markedly influences trafficking to the cell surface PM [100,101,102]. Although the canonical GαS/cAMP/PKA signaling pathway has been accepted for a long time as the effector mechanism of gonadotropin biological action, it is currently known that gonadotropin receptors (and the TSHR as well) may couple to other G protein subtypes and activate a number of distinct signaling pathways [94,103], depending on the cell context and developmental stage of the host cells [104].

Gonadotropin receptors bear several structural motifs and posttranslational modifications that regulate their folding and intracellular traffic to the PM. These features include several motifs present in distinct domains as the AFNGT sequence motif in the ECD (amino acid residues 193 to 197 in the LHCGR and 189 to 193 in the FSHR), which contains a potential glycosylation site [N191GT and N195GT, in the FSHR and LHCGR, respectively]. Mutations in this motif influence receptor folding and trafficking to the PM, and thereby cause diseases characterized by resistance to gonadotropins [102,105,106]. Carbohydrates in the ECD of both gonadotropin receptors, are not involved in hormone binding, but represent key structures for the maturation process of the newly synthesized receptors, promoting their correct folding, conformational stability, and trafficking to the PM [102]. Naturally occurring mutations in the ECD of the human FSH and LHCG receptors near or at those putative glycosylation sites confirms the critical role of glycosylation in targeting of the gonadotropin receptors to the PM [107]. Other motifs of the gonadotropin receptors than are involved in folding and trafficking and thereby exit of the receptors from the ER include the E/DRY motif (ERW in the gonadotropin receptors) at the boundary of the TMD3 and the IL2 and the N/DPxxY motif (NPFLY in the gonadotropin receptors) at the TMD7 near the cytoplasmic face of the PM and that are fundamental for the structure and function not only for these but also for other GPCRs [25].

**Figure 4:**
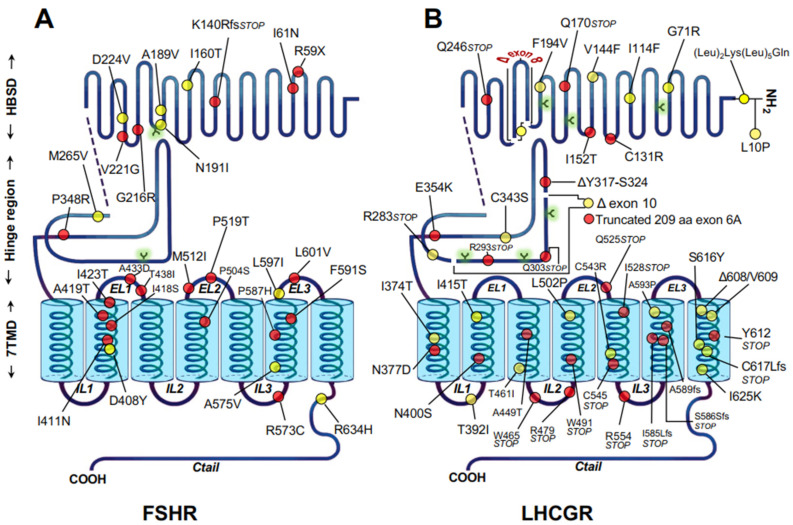
Schematic representation of the human gonadotropín receptors showing the location of the inactivating mutations described to date (red and yellow circles). Mutations indicated by the yellow circles lead to misfolding and trafficking defective receptors with reduced or absent cell surface plasma membrane expression. Arbor-like structures (shaded in light green circles) represent potential glycosylation sites. (**A**) FSHR; (**B**) LHCGR.

As with the GnRHR, mutations resulting in changes in protein sequence of gonadotropin receptors may lead to loss-of-function of the receptor, leading to hypogonadism in humans. A number of naturally occurring, inactivating mutations of the *LHCGR* and *FSHR* associated with particular reproductive disorders have been reported (Figure 4) [42,102,108]. These mutations frequently lead to disease when both alleles are affected by a mutation, as observed in individuals who are homozygous or compound heterozygous. Inactivating mutations of the gonadotropin receptors are germline, missense or nonsense mutations that result in single amino acid substitutions in the receptor protein or introduction of a stop codon in their corresponding mRNAs, leading to amino acid deletions or insertions, or premature truncations of the protein (Figure 4A,B). Due to the scattered distribution of the mutations along the sequence of the receptor, the mutations may compromise the synthesis of the receptor protein due to large truncations (Class I GPCR mutations), regions involved in ligand binding (Class III mutations) or receptor activation or domains involved in G protein coupling (Class IV mutations). The mutations may also lead to misfolding resulting in trafficking defective proteins which are unable to correctly route to their final destination (i.e., the cell surface PM) (Class II mutations) [13] (Figure 4). These functional defects are not mutually exclusive, as one mutation may cause functional defects on both intracellular traffic and any other function. Loss-of-function mutations in the gonadotropin receptor genes and their clinical consequences have been previously described extensively (reviewed in [42,109]). In general, the clinical phenotype of patients bearing these mutations vary depending on the degree of residual function of the mutant receptor, which is an important determinant for the response to exogenous gonadotropins (when expressed at the PM) or to potentially useful pharmacochaperone drugs, as may occur in Class II mutations (see below). In addition to causing defective folding and intracellular retention of the protein, Class II mutations in the gonadotropin receptors may also affect an intrinsic function of the receptor (e.g., binding to agonist or signal transduction); thus, it might be expected that the benefit of treatment with pharmacoperones to correct folding and trafficking will be limited. Nevertheless, the possibility exists that the pharmacoperone may correct not only trafficking and routing of the receptor but also receptor function by virtue of modifying the conformation affected by the mutation that primarily impacts ligand binding or signal transduction. This effect, in fact, has been reported for the cell-permeant, allosteric small molecule agonist Org 42,599/Org 43,553, which corrected the function of the binding deficient C131R and I152T, or the E354T signaling deficient LHCGRs (Figure 4) [52,110].

Among the ~40 loss-of-function LHCGR mutants described to date, approximately 40% are nonsense or frameshift mutations and at least half are trafficking defective receptors in which the net number of functional receptors expressed at the cell surface PM is decreased to a variable extent [102,110]. The phenotype of individuals harboring these inactivating mutations of the LHCGR leading to misrouting was, therefore, defined by the density of residual receptors with preserved function that were not retained intracellularly by the QCS and that reached the PM and bound agonist. As shown in Figure 4B, the functionally characterized, trafficking defective LHCGRs bear mutations either in their ectodomain or hinge region.

Some of these trafficking defective mutant LHCGRs are interesting from structural and functional points of view. In the F194V misfolded mutant, the substitution is located at a highly conserved motif of the gonadotropin receptors (193AFNGT197, in the LHCGR) which contains the aforementioned NGT glycosylation motif. This mutation severely impairs trafficking of the mutant receptor to the PM without altering agonist affinity [105]. Both the A593P and S616Y loss-of-function mutants exhibit normal agonist binding affinity, but their ability to activate intracellular signaling is severely impaired due to misfolding and intracellular retention of the majority of mutant receptor synthesized [111,112]; these mutants have distinct conformations and exhibit different folding configurations during their maturation process at the ER, as indicated by their differential association with molecular chaperones [112]. Extensive deletions, as those in exons 10 and 8, which are within the putative LRR and hinge regions of the LHCGR, may severely affect receptor conformation due to misfolding and lead to impaired ability of the protein to interact with its cognate ligand and activate.

Naturally occurring mutations of the *FSHR* are fewer in number but can be similarly classified. In fact, only ~30 inactivating mutations in the hFSHR have been reported to date (Figure 4A) [42,108,113,114,115], of which at least nine are trafficking-defective proteins, many of them identified as intracellularly retained molecules by different techniques including fluorescence microscopy and flow cytometry. As it has been observed with the inactivating LHCGR mutations, there is in general a good correlation between residual activity exhibited by the mutant FSHRs and the spectrum of the clinical phenotype expressed by the patients bearing the mutation(s) [106,107,116], ranging from congenital, complete ovarian failure, to premature ovarian failure in adult life. Interestingly, the phenotype in homozygous men is not clinically defined, as fertility is preserved despite altered sperm quality [117]. The most severe phenotype is exhibited by females homozygous for the trafficking-defective loss-of-function FSHR mutations A189V, P519T, and D408Y, who presented with hypergonadotropic hypogonadism, arrest of follicular maturation beyond the primary stage and complete lack of responsiveness to FSH [106,107,118].

The naturally occurring mutation A189V causes a profound defect in targeting the receptor protein to the PM [119], as it compromises integrity of the 189AFNGT193 glycosylation motif. Valine in position 189 as well as isoleucine 191 (in the LHCGR) may interfere with the structural integrity of the LRRs, which host the glycosylation site, and alteration of this structure may impair receptor LRR folding, particularly its α-helical portion. Although the loss of a putative glycosylation site may affect, per se, folding and trafficking of the mutant receptor to the PM, it is not known whether the A189V mutant is glycosylated or not at N191. Overexpression of the A189V in vitro revealed that a negligible amount of the mutated receptor was present at the PM and most of the receptor protein appeared sequestered and retained inside the cell [119]. Further, the reduced level of PM expression of the A189V FSHR mutant confers preferential coupling to the β-arrestin-mediated ERK 1/2 signaling pathway, similar to that observed when the WT receptor is expressed at low PM levels [120]; this finding indicates that the selective signaling observed is due to the low density of PM expression of the receptor rather than the mutation causing a functional defect. In the case of the N191I mutant [121], it is also possible that its limited PM expression may be due to alterations in the structural integrity of the ectodomain at the 189AFNGT193 glycosylation motif rather than to the absence of glycosylation at this particular site. The FSHR P519T mutation in the center of the EL2 leads to complete failure to bind agonist and trigger intracellular signaling. It seems that the loss of a proline at position 519 provokes a severe conformational defect that causes trapping of the receptor at the ER [118] due to the loss of the forced turn in the protein structure by the substitution with the more reactive threonine; it is thus possible that the abrupt turn in the middle of the EL2 is probably a requisite not only for activity but also for routing.

**Figure 5:**
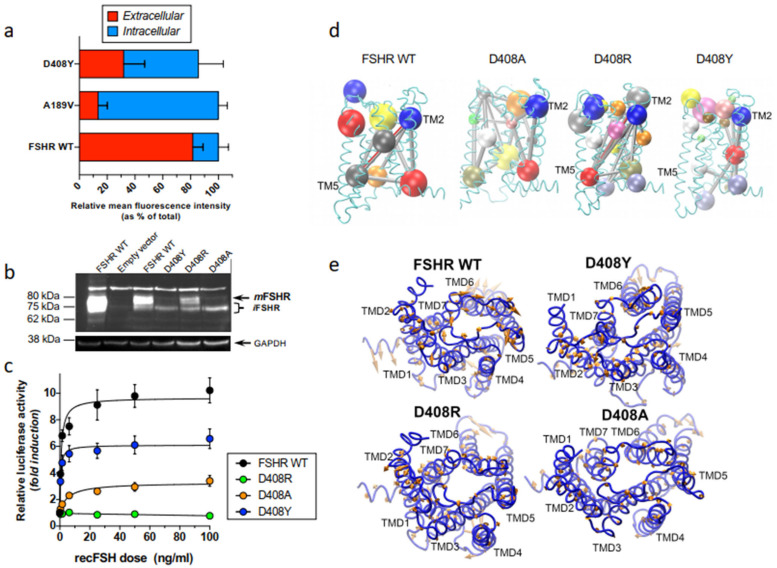
In vitro and in silico features of the misfolded, trafficking-deficient, naturally occurring D408Y FSHR mutant and other laboratory-manufactured FSHRs with distinct substitutions at position 408 of the receptor protein. (**a**) Extra- and intracellular mean relative fluorescence intensity of the WT and D408Y FSHRs expressed in HEK-293 cells as disclosed by flow cytometry. Note the limited cell surface plasma membrane expression of the mutant receptor. The figure also shows data on the trafficking deficient A189V FSHR mutant included for comparison. (**b**) Representative Western blot of the WT FSHR and the D408Y, D408R, and D408A mutant FSHRs present in extracts of HEK-293 cells transiently transfected with each receptor species (lanes 3–6). The immunoblot shows the relevant portion of an autoradiogram in which the mature (*m*FSHR, ~80 KDa), plasma membrane-expressed, fully glycosylatyed form of the D408Y and D408A FSHRs are considerably reduced. These two latter FSHR mutants are predominantly detected as immature, intracellular forms of the receptor (*i*FSHR, KDa ≤ 75). Lane 1: WT FSHR from extracts of HEK-293 cells stably expressing the human FSHR; lane 2: Extracts from cells transfected with empty vector. (**c**) Dose-response curves for pSOMLuc expression by HEK-293 cells transiently cotransfected with the WT or mutant D408Y, D408R, or D408A FSHRs and the cAMP-sensitive pSOMLuc reporter plasmid, and exposed to increasing doses of recombinant human FSH (recFSH). Each point represents the mean ± SEM of 3 independent experiments. Note that the D408R and D408A FSHR mutants are completely or partially inactive when challenged with recFSH, whereas the function of the naturally occurring D408Y mutant also exhibits reduced activity albeit to a lesser extent than the other mutants. (**d**) Dynamic community analysis of the WT, D408Y, D480A, and D408R FSHRs. When compared with the WT FSHR, distinct connectivities among communities were observed in the mutant receptors. (**e**) Principal component analysis (PCA) to evaluate the collective motion of Cα atoms in the WT and D408Y, D408R, and D408A FSHRs. In these three-dimensional representations, it can be observed that the dynamics of the receptor disclosed differences in the amplitudes of interhelical domains (arrows) as a function of the mutation at position 408. See the text and Refs. [115,122] for data and details.

We have recently employed computational modeling and molecular dynamics simulations (MDS) of the FSHR in an explicit membrane environment [pre-equilibrated lipid bilayer of 1-stearoyl-2-docosahexaenoyl-sn-glycero-3-phosphocholine (SDPC) molecules] as a tool to explore in more detail the impact of the substitution of aspartate with tyrosine, alanine, or arginine at position 408 [122]. The FSHR modelled allowed us to detect differences in the receptor dynamics at the transmembrane domains between the WT and mutant receptors, likely related to receptor function. The D408Y mutation is a misfolded mutant that severely affects trafficking and function of the receptor molecule, and ~70% of total protein accumulates intracellularly [115,123] (Figure 5a). This mutant is mainly expressed as an immature receptor as disclosed by immunoblotting (Figure 5b), and exhibits substantially reduced cAMP-sensitive activation of a CRE (cAMP response element)-controlled firefly luciferase reporter gene (Figure 5c). Dynamic community analysis of the trafficking-defective D408Y FSHR, which is a calculation over the covariance matrices when highly correlated atoms are grouped together in interconnected communities, revealed a distinct connectivity among communities in this mutant (Figure 5d). Comparison between the WT and mutant D408Y receptor revealed the presence of the interhelical connectivity between the extracellular side of TMD 1 and the intracellular side of TMD6 of the WT receptor whereas in the D408Y mutant this interhelical connectivity was virtually lost. Two additional mutants were also analyzed for comparison, the D480A and D408R mutant FSHRs. Although both of these mutants also were severely dysfunctional, (Figure 5c) membrane expression of the mature, PM-expressed form of the D408R mutant was clearly detected by immunoblotting (Figure 5b). Nevertheless, in the D408A receptor, disruption of side chain interactions and conformational dynamics was revealed by the loss of interhelical connectivity (Figure 5d). Furthermore, we performed principal component analysis (PCA) to evaluate the collective motion of Cα atoms [124]. From the projection of simulation trajectories along few eigenvectors, the resultant principal modes provided the collective motion that included the highest amount of the total fluctuation. In Figure 5e, projections of the first principal mode are shown for the WT FSHR and the mutants D408Y, D408R, and D408A. It can be observed that the dynamics of the receptor disclosed interesting differences in the amplitudes of interhelical domains as functions of the mutation at position 408. For example, atom displacements of the helical domains showed lower amplitudes for the D408Y and D408A mutants than those observed for the WT or D408R FSHRs. These data indicated that the global dynamics of the FSHR was sensitive to mutations at amino acid residue 408 specifically to the electrostatics generated by the aspartate acidic residue, and that mutations at position 408 impacted on proper routing and function of this receptor.

## 3. Strategies to Rescue Misfolded GPCRs Leading to Endocrine Disorders

### 3.1. General Approaches to Rescue Function of Misfolded Proteins

Several strategies to correct folding and to promote trafficking of misfolded proteins to the PM have been described [6,8,20,21]. These include physical, chemical, genetic, and pharmacological approaches. Among the physical strategies, it has been shown that incubation of cells expressing mutant misfolded membrane proteins at low temperatures (20–30 °C) promotes PM expression and in some cases normal function whenever the mutation does not compromise critical residues involved in function. For example, studies on the biosynthesis of the cystic fibrosis transmembrane conductance regulator (CFTR) F508 deletion mutant (which leads to cystic fibrosis), showed that the mutation leads to misfolding and ER trapping of the mutant protein [125,126,127]. Incubation of cells expressing this particular mutant at low temperatures, reverted processing of the CFTR mutant towards the WT species, promoting membrane expression of the channel and normal function [128]. Likewise, increased PM expression of several conformationally defective α_2C_-adrenergic (e.g., α_2C_322–325del), GnRH (N10K, R262Q, Y284C, and Q106R), LHCG (K541A, K544A, K547A, and T175A) and FSH (D408Y) mutant receptors resulted from incubating cells bearing the mutant receptor at lower (26–32 °C) temperatures, apparently through a mechanism involving inhibition of HSP90 activity on receptor traffic [8,115,129,130]. Thus, for some misfolded proteins that are temperature-sensitive, the use of physical methods may counteract their retention at the ER by the QCS and facilitate trafficking of the abnormal protein to their physiological site of action.

Another strategy for enhancing cell surface PM expression of misfolded proteins is by introducing genetic modifications into the sequence of the conformationally abnormal protein (i.e., genetic rescue) [72,131,132]. This approach allows abnormal proteins bearing genetic defects to be overexpressed or stabilized and thus it does not provoke global changes in the ER secretory activity. For example, in the case of the mammalian GnRHR, which lacks the Ctail extension, insertion of this domain from other species (e.g., fish) or deletion of K191 (which restricts PM expression), dramatically increases PM expression in both cases (Figure 1) [59,72,133]. Nevertheless, genetic strategies are quite impractical as therapeutic intervention because the primary error could be directly corrected by accessing the gene sequence. In vitro rescue of misfolded membrane receptors can also be accomplished by manipulating ER and/or post-ER mechanisms that regulate protein export, through controlling synthesis or expression of molecular chaperones involved in protein trafficking (e.g., calnexin, BiP/Grp78) or by employing cell-penetrating peptides that modify cytosolic Ca^2+^ stores, thereby affecting function of Ca^2+^-regulated chaperones as those involved in post-ER quality control [134,135,136]. Nevertheless, as in the case of chemical chaperones (see below), the major problem of these approaches is their lack of specificity for the target protein.

### 3.2. Chemical Approaches

In chemical rescue, cell surface PM expression (and function) of misfolded proteins can be achieved by incubating cells expressing the mutant protein with non-specific stabilizing agents (e.g., polyols and sugars) [6,137]. These agents or chemical chaperones are small molecular weight compounds that promote protein folding by stabilizing their conformation without interacting with the protein or interfering with their function. Osmolites stabilize proteins by reducing the free movement of proteins as well as by increasing their hydration [137], preventing aggregation of intermediate conformers and promoting protein stabilization through modifying the free-energy difference between partially folded and more compact native structures. Since chemical chaperones require high concentrations for effective folding of mutant proteins, they are too toxic for in vivo applications. In addition, although chemical chaperones can rescue some misfolded proteins, their effects may be dangerous in that they are nonspecific and might potentially lead to increased secretion or intracellular retention of a variety of proteins, compromising cell function [6], albeit with a few exceptions [138].

### 3.3. Pharmacological Approaches

In contrast with chemical chaperones, pharmacological chaperones have the advantage of selective binding to the conformationally defective protein without interfering with the degradation of other misfolded proteins that require elimination from the cell as part of the normal process of proteostasis [6,13,18,32,34,48,49,139]. Pharmacological chaperones can also interact with oligomerized receptors in which one of the monomers is misfolded and exerts quaternary interactions with the wild-type receptor species acting as the dominant negative, thereby limiting its cell surface delivery to the plasma membrane [140,141]. Pharmacochaperones or pharmacoperones are small, PM permeable molecules (that act as agonists or antagonists of the natural ligand, or as allosteric modulators) that penetrate cells and serve as a molecular scaffold to promote correct folding and prevent aggregation of misfolded proteins within the cell [8,18,22,23,32,48,142,143,144]. The efficiency of pharmacoperones to rescue PM expression and function depends on several factors, including the particular structure of the pharmacoperone (which determines selectivity toward the target protein), the severity of the folding defect present in the target protein, and the specific location of the mutation [34]. For example, mutant human V_2_Rs displaying amino acid exchanges at the interface of the TMD2 and TMD4 (H80R, W164R, and S167L mutants) are resistant to pharmacoperone treatment probably because the severe folding defect imposed by the replacing residues [145]. As discussed above, in the case of the S168R and S217R hGnRHR mutants (Figure 1), replacement of any of these serine residues by the highly hydrophilic arginine provokes a thermodynamically unfavorable exchange that rotates TMDs 4 and 5, displacing the EL2, and making difficult the formation of the C14–C200 bridge [64] (Figure 1, Figure 2 and Figure 3). As mentioned earlier, the C14–C200 disulfide bridge of the hGnRHR stabilizes the receptor in a conformation compatible with ER export. Consequently, both serine → arginine mutants are completely resistant to pharmacoperones in vitro [10,70].

A number of endocrine diseases, including primary hypothyroidism and obesity, X-linked nephrogenic diabetes insipidus, and reproductive disorders, among others, may be caused by misfolded GPCR mutants [9,39]. Some of these mutant GPCRs have been reported to show functional rescue with different pharmacoperone compounds in in vitro and in vivo systems (Table 1). X-linked nephrogenic diabetes insipidus (NDI) in humans is frequently caused by misfolded, traffic-defective mutants of the V_2_R. It has been shown that distinct cell membrane permeable antagonists may rescue function in vitro of several of these mutant V_2_Rs [20]. For example, the nonpeptide antagonists SR49059, OPC31260, OPC41061 (tolvaptan), and SR121463B (satavaptan) were able to induce maturation and basolateral membrane localization and function in eight out of nine V_2_R mutants in stably transfected MDCK cells [50]. Another example is the aminoglycoside antibiotic (G418) which rescued a V_2_R mutant with a premature truncation causing X-linked NDI [146]; addition of this antibiotic to kidney collecting duct cells expressing the E242X V2R mutant increased arginine vasopressin-stimulated cAMP responses. In mutant E242X mice, the G418 compound suppressed the premature stop codon, facilitating protein translation to continue the normal end of the gene, leading to improved urine-concentrating ability [147]. In an in vivo proof-of-principle study, the effect of the peptidomimetic V_1A_R/V_2_R antagonist SR49059 to rescue function of R137H, W164S, and des185-193 V_2_R mutants in patients with NDI was studied [20]. In this seminal study, a significant drop in urine production and water intake concurrent with a significant increase in urine osmolarity in response to this particular compound occurred in a subset of patients with NDI. Clinical rescue of functional mutant V_2_Rs at clinically safe, feasible concentrations has been reported to be most effective with OPC31260 and OPC41061; these compounds are promising candidates to ameliorate the alterations of NDI [49]. As in the case of the TMDs 4 and 5, S168R and S217R hGnRHR mutants (Figure 1), V_2_Rs displaying amino acid exchanges at the interface of the TMDs 2 and 4 (H80R, W164R, and S167L mutants) were resistant to pharmacoperone-mediated cell surface delivery, probably due to the severe folding defect provoked by the replacing residues [49,50,148], emphasizing the importance of the particular location of the mutation and the nature of the compound on the response of misfolded V_2_R mutants and other GPCRs, as well as to pharmacoperone drugs. Another example are several MC4R mutants which lead to monogenic obesity; some the mutant MC4Rs are misfolded proteins retained intracellularly [37,38,40]. Mutants of this GPCR, which leads to severe early-onset morbid obesity in humans, may be rescued by Ipsen 5i [55], a pharmacological chaperone that has been demonstrated to be a novel, potent drug capable of correcting trafficking and signaling in a significant number (73%) of intracellularly trapped mutants [9,55]. Another small molecule pharmacoperone is ML00253764; as with Ipsen 5i, ML00253764 is a MC4R antagonist and a partial inverse agonist able to cross the blood-brain barrier [149,150] that increases cell surface expression of mutant receptors [38,151]. THIQ is a small molecule agonist that also can penetrate the blood-brain barrier [152]; this compound shares part of the binding pocket with [Nle^4^, DPhe^7^]-α-MSH (*NDP*-*MSH*), a potent α-MSH analogue, and exhibits a more efficient pharmacoperone activity in neuronal cells [153], expressing several mutant MC_4_Rs in Neuro2a and NIE-115 neuronal cell lines [152]. Mutations in the calcium-sensing receptor (CaSR) cause calcium-handling diseases [154,155]. Allosteric agonists, such as NPS R-568, have been shown to stabilize CaSR mutants. NPS R-568 is a cotranslational stabilizer acting at a conformational checkpoint during CaSR biosynthesis [154,156]. A number of CaSR Class II loss-of-function mutants leading to familial hypocalciuric hypercalcemia and neonatal hyperparathyrodism can be stabilized and rescued by NPS R-568 [157].

As mentioned previously, several GPCRs participate in the regulation of reproductive function at different levels. These receptors include the NK3R and KISS1R, which play an essential role in the function of the hypothalamic GnRH pulse generator, the PROKR2, involved in the migration of GnRH-producing neurons from the olfactory bulb to the hypothalamus during embryogenesis, and the above discussed GnRH, follicle-stimulating hormone, and luteinizing hormone receptors, which are more directly involved in the regulation of gonadal function [44,45,46,111,114,119,132]. Several pharmacoperones for the later GPCRs leading to congenital hypogonadism due to receptor misfolding (PROKR2, GnRHR, FSHR and LHCGR) have been previously described [42,51,52,53,71,158].

Kallmann syndrome is a disease characterized by the presence of hypogonadotropic hypogonadism and anosmia/hyposmia, causing delayed puberty and infertility. Among the mutations leading to this disease are those involving the PROKR2, some of which provoke misfolding and intracellular trapping of the receptor. A small molecule, A457 (a PROKR2 antagonist), was found to rescue, to some extent, cell-surface plasma membrane expression and signaling of intracellularly retained PKR2 mutants [158]. Several small molecules (including the indole IN3, the quinolone Q89 and two erythromycin macrolides, A177775 and TAK-013, originally developed as GnRH peptidomimetic antagonists) that rescue misfolded human GnRHRs have been described in some detail [71,159]. These pharmacoperones form a surrogate bridge between residues D98 (at the extracellular face of TMD 2) and K121 (at the TMD 3) to replace the naturally occurring E90-K121 salt bridge (Figure 1), thus promoting trafficking of GnRHR mutant to the plasma membrane [160]. Pharmacological rescue (assessed by ligand binding and restoration of receptor coupling to effector) of five naturally occurring GnRHR mutants (T32I, E90K, C200Y, C279Y, and L266R) identified from patients with HH was demonstrated in transiently transfected Cos-7 cells, whereas in vivo rescue by the indole IN3 was demonstrated using a mouse model bearing the GnRHR E90K mutation [161]; a similar in vivo study illustrated the efficacy of pharmacoperone therapy through the analysis of changes in several physiological and biochemical biomarkers after pulsatile administration of the indole pharmacoperone IN3 to mutant male mice [73,74].

The advantage of pharmacoperones acting via allosteric interactions with the misfolded receptor is that allosteric compounds *do not compete* with the natural agonist for the binding site. For example, in the case of the misfolded A189V human FSHR, in vitro exposure of Cos-7 cells expressing this mutant receptor to the thienopyr(im)idine Org41841 resulted in almost a twofold increase in PM expression and FSH-stimulated cAMP production without significantly altering mRNA expression of the receptor or its ligand binding affinity [54]. A more recent in vitro study on several misfolded human FSHRs leading to primary hypogonadism employed the dihydrobenzoindazole small molecule analogue CAN1404 previously described by Organon in the published patent WO2011/012674 [53]. Of the eleven FSHR mutants with severely reduced cell surface expression, significant rescue was achieved for six by treatment with CAN1404 for 24 h and a corresponding increase in FSH-induced signaling was observed for four of these misfolded mutants, indicating functional recovery [53]. Similarly, incubation of cells expressing the misfolded LHCGR mutants A593P and S616Y (which lead to Leydig cell hypoplasia and varying degrees of genital ambiguity) with the cell-permeant, allosterically binding small molecule agonist Org 42599, led to rescue of PM expression and signaling of the two LHCGR misfolded mutants [51,52]. Thus, pharmacoperone strategy is a promising therapeutic approach feasible for treating endocrine and nonendocrine diseases caused by misfolded GPCRs.

## 4. Conclusions

We have herein briefly discussed how mutations in GPCRs involved in endocrine function may affect misfolding and targeting of newly synthesized receptor proteins to the cell surface plasma membrane. As discussed in this review, misfolding as a result of mutations in the sequence of the receptor leads to production of conformationally abnormal proteins that are retained in the ER by the QCS of the cell. Mutations in GPCRs leading to misfolding and misrouting are more frequent than originally thought, and, for a number of GPCRs, there is currently considerable information on the molecular mechanisms whereby misfolded GPCRs lead to disease.

A number of endocrine diseases are caused by misfolded GPCR receptors, including those critical for the adequate function of the hypothalamic-pítuitary-gonadal axis. Several PM permeable small molecules (agonists, antagonists, and allosteric modulators) have been tested in vitro and in vivo for their effects on rescuing expression and function of misfolded receptors intracellularly retained by the QCS [9,13,20,50,51,53,74,85,156,162,163,164,165]. In this regard, a number of high-throughput screens (HTS) have been designed to identify new small molecules that may act as pharmacoperone drugs for the treatment of an array of protein conformational disorders, including those involving GPCRs, enzymes, ion channels, and lysosomal storage [13,163,166]. HTS is a valuable, cost-effective means for rapid testing, detection of hits for different diseases, and evaluation of toxicity prior to testing in clinical settings.

## Figures and Tables

**Table 1:** Loss-of-function diseases caused by folding defects in GPCRs associated with endocrine function and pharmacological chaperones to correct folding and trafficking in vitro or in vivo.

Disease	GPCR	Pharmacoperone
Nephrogenic diabetes insípidus	V2R	Satavaptan, relcovaptan, VPA985, YM087, tolvaptan, OPC31260 [20,48,49,50]
Isolated hypogonadotropic hypogonadism	GnRHR	IN3, IN30, Q89, A177775, TAK-013 (reviewed in Ref. [22])
Familial hypocalciuric hypercalcemia	CaR	NPS R-568 (reviewed in Ref. [36])
Male pseudohermaphroditism and hypergonadotropic hypogonadism	LHR	Org 42,599 [51,52]
Primary ovarian failure	FSHR	Org41841, CAN1404 [53,54]
Congenital hypothyroidism	TSHR	-
Familial glucocorticoid resistance	MC2R (ACTHR)	-
Obesity	MCR3, MCR4	ML00253764, Ipsen 5i, THIQ [9,13,38,39,55]

## Data Availability

Not applicable.
